# Spectral theory of spin relaxation

**DOI:** 10.1098/rsos.260478

**Published:** 2026-11-04

**Authors:** Timothy Field

**Affiliations:** Electrical & Computer Engineering and Mathematics & Statistics, McMaster University, Hamilton, Ontario, Canada

**Keywords:** fluctuations and noise, non-Markovian processes, quantum spin models, stochastic differential equations, spin relaxation, nuclear magnetic resonance, Lorentzian spectra, electron paramagnetic resonance, random magnetic fields, density matrix formalism

## Abstract

A dynamical theory of spin relaxation previously formulated in terms of stochastic differential equations is extended beyond the extreme narrowed approximation to incorporate temporal correlation in the driving random magnetic fields. An analysis of spin–spin relaxation in this case is provided, which is non-perturbative in the random field and yields an exact solution to the spin dynamical equations for axial (longitudinal) field fluctuations. We provide a real-time dynamical derivation that leads to a generalized class of spin spectra that are essentially non-Lorentzian for all finite field parameters. A spectral-moment analysis and novel parameter-extraction scheme lead to distinct notions of observable relaxation timescales compared with the standard characteristic exponential. The Lorentzian case is recovered as the extreme narrowed limit.

## 1. Introduction and context

Previously, in [1], a dynamical theory of spin relaxation was proposed which assumed the extreme narrowed approximation for the driving random magnetic fields. To recap the main ideas of that earlier work, the dynamics of spin relaxation was formulated from the point of view of stochastic differential equations, as a real-time process for an ensemble of spins immersed in random magnetic fields, as opposed to the standard density matrix approach, which represents only the ensemble-average behaviour. In doing so, it was possible to capture the so-called ‘spin-noise’ process in terms of its dynamical origins, and identify this as the basis of standard relaxation through the statistical expectation, whereby a standard free induction decay is mirrored as an autocorrelation function in a real-time dynamical theory. The central result of that paper is a stochastic differential equation (SDE) for the combined processes of spin–spin and spin–lattice relaxation, expressed in terms of the modified spin density (MSD). That MSD is an object introduced to capture the real-time non-ensemble-averaged properties of a spin system, which differs from the ordinary density matrix in terms of its scaling with the number of spins.

Specifically, by way of context for the present new results, we provide a brief summary of the previous model framework. For a population of size N, we construct an MSD as a suitably scaled sum over projection operators corresponding to constituent random *pure* states of the system $\left\{|\sigma_{i}\rangle \right\}$:

(1.1) $$\Sigma_{t}=\frac{1}{N^{1/2}}\sum_{i}|\sigma_{i}\rangle \langle \sigma_{i}|.$$

This differs from the ordinary density matrix via the square-root reciprocal scaling with respect to the number of spins, in place of reciprocal N. Accordingly, the MSD captures *non*-ensemble-averaged properties of the quantum mechanical (spin) ensemble [1]. For the ensemble, in relation to equation (1.1) and the main result that we shall recall presently in this section, we may write componentwise the matrix

(1.2) $$\Sigma_{t}=\left(\begin{array}{cc} \Sigma_{t}^{↑↑} & \Sigma_{t}^{↑↓}\\ \Sigma_{t}^{↓↑} & \Sigma_{t}^{↓↓} \end{array}\right).$$

### 1.1. Random Hamiltonian

The unperturbed Hamiltonian is given by $H_{0}=-\mu \cdot B_{0}$ (taking due note of the minus sign), where the magnetic moment is μ=γs (γ is the gyromagnetic ratio) for spin $s=\frac{1}{2}\hbar \sigma$, in which $\left(\sigma_{x},\sigma_{y},\sigma_{z}\right)$ are the Pauli matrices. In matrix notation, we then have

(1.3) $$H_{0}=\left(\begin{array}{cc} E & 0\\ 0 & -E \end{array}\right).$$

This implies a negative energy eigenvalue $E_{↓}<0$ for $B_{0}$ in the positive z-direction and thus the spins tend to align *parallel* to the magnetic field (cf. eqn (83) and the discussion of §IIIE in [1]). In what follows, we shall consider a time-dependent random perturbed Hamiltonian

(1.4) $$H(t)=H_{0}+H_{1}(t)=-\gamma s\cdot B(t),$$

in which B(t) is the effective random magnetic field environment experienced by an individual (proton) spin. This consists of the main field $B_{0}$ plus a (rapidly) fluctuating field $B_{1}(t)$ corresponding to the rapid (rotational) motion of the spin (nucleus) in its magnetic environment. We shall not be concerned with the specific interaction, e.g. dipole–dipole, chemical shielding and quadrupolar, that gives rise to the effective field fluctuations, and for the purpose of this summary, here we describe the case of isotropic fluctuations in the ‘extreme narrowing’ approximation where the autocorrelation time for $B_{1}(t)$ is extremely small, indeed negligible.

It is appropriate at this point to recount Abragam’s treatment of the master equation for the density matrix [2, p. 276]. Abragam begins with the equation of motion for the density matrix in the presence of a perturbed Hamiltonian

(1.5) $$\frac{1}{i}\frac{\text{d}\sigma }{\text{d}t}=-[H_{0}+H_{1}(t),\sigma ],$$

in which the perturbation $H_{1}(t)$ is a stationary operator-valued random process. Then, in the interaction representation defining ‘∗’ as $O^{*}=e^{iH_{0}t}Oe^{-iH_{0}t}$ for any operator O, Abragam [2] observes

(1.6) $$\frac{1}{i}\frac{\text{d}\sigma^{*}}{\text{d}t}=-[{H_{1}}^{*}(t),\sigma^{*}],$$

which can be integrated in a perturbation expansion in the perturbing Hamiltonian $H_{1}$, assumed to be small (see [2] and also eqn (29) in [1]). Our previous approach considered a perturbation involving fluctuations in all directions:

(1.7) $$\begin{array}{rl} H_{1}(t) & =\frac{1}{2}k^{\frac{1}{2}}\sigma \cdot \left(\Gamma_{t}^{\left(x\right)},\Gamma_{t}^{\left(y\right)},\Gamma_{t}^{\left(z\right)}\right)\\ & =\frac{1}{2}k^{\frac{1}{2}}\left(\begin{array}{cc} \Gamma_{t}^{\left(z\right)} & \Gamma_{t}^{\left(x\right)}-i\Gamma_{t}^{\left(y\right)}\\ \Gamma_{t}^{\left(x\right)}+i\Gamma_{t}^{\left(y\right)} & -\Gamma_{t}^{\left(z\right)} \end{array}\right), \end{array}$$

in which $\Gamma^{\left(\cdot \right)}$ is a three-dimensional δ-correlated (Gaussian) white noise process, thereby consistent with the extreme narrowed approximation. If a background magnetic field is present, then the Zeeman interaction gives rise to a Boltzmann equilibrium spin population distribution, split between the energy levels at temperature T, for spins ↑ and ↓ according to the Boltzmann ratio $N_{↑}/N_{↓}=e^{-\Delta E/kT}$, for $\Delta E=E_{↑}-E_{↓}$. Defining $p_{↑}:=\frac{N_{↑}}{N_{↑}+N_{↓}}=\frac{1}{1+N_{↓}/N_{↑}}$, we deduce the expression for the longitudinal polarization (Boltzmann) fraction:

(1.8) $$p_{↑}=\frac{1}{1+\exp(\Delta E/kT)},$$

so that for a field aligned along positive z, we have ΔE<0 and therefore p>1/2. In this model framework, we now recall our main previous result as follows (eqn (92) in [1]).

*The combined processes of spin–spin and spin–lattice relaxation are determined by the following set of stochastic differential equations for the modified spin density:*

(1.9) $$\begin{array}{c} \text{d}\Sigma_{t}=\left(\begin{array}{cc} -k(\Sigma_{t}^{↑↑}-\frac{1}{2}N^{1/2}) & -k\Sigma_{t}^{↑↓}\\ {}* & - \end{array}\right)\text{d}t\\ +\left(\begin{array}{cc} -\sqrt{{}^{k}/{}_{6}}\;\text{d}W_{t} & \sqrt{{}^{k}/{}_{3}}\;\text{d}\xi_{t}\\ {}* & - \end{array}\right)\\ +\frac{1}{2}k(1-2p)N^{1/2}\left(\begin{array}{cc} -1 & 0\\ 0 & 1 \end{array}\right)\text{d}t. \end{array}$$

(Notationally, the symbols ‘∗’ and ‘−’ in the bottom rows of the first two matrices appearing on the right-hand side of the above equation denote the complex conjugate and negative of the elements diagonally opposite, respectively.) As derived previously in §III D5 of [1], the fluctuating Wiener terms $\text{d}W_{t}$ and $\text{d}\xi_{t}$ arising in equation (1.9) are statistically independent, being associated with the longitudinal and transverse parts of the relaxation, respectively.

These results were explored further in [3] in terms of real-time measurements, including non-stationary behaviour. More recently, a study was made of the corresponding stochastic dynamics of *entangled* spins immersed in (spatially) correlated random fields with a variety of assumptions concerning the inter-field correlation, which served to identify relaxation and disentanglement timescales and their asymptotic density matrix for an ensemble of spins [4]. Again here, it will be relevant to consider a more general type of random field that is temporally correlated, in order to make closer contact with experimental situations. Also of note in this context are techniques for spin-noise imaging [5] for which a dynamical theory is relevant. Some notable mathematically related work in applications to lasers, using non-Lorentzian line shapes, exists in the established literature [6,7]. Other data-driven approaches which exploit the entirety of the autocorrelation function, as opposed to a finite set of relaxation parameters, can be important in very specific experimental situations, and for which there is an extensive literature (e.g. [8–14]). Nonetheless, the Lorentzian spectrum is still prevalent as a generic benchmark in textbook treatments and for a broad range of nuclear magnetic resonance (NMR)/electron paramagnetic resonance (EPR) applications, and accordingly our purpose here is to propose a natural and tractable generalized alternative that incorporates the temporal correlation properties of random fields, which are always present in laboratory experiments. Thereby, some of the distinctly unphysical features of the Lorentzian, in particular the apparent paradox of the infinite spectral variance that it predicts, are neatly resolved, as will readily be seen in terms of the new properties of spectral moments.

The paper is organized as follows. In §2, we develop an extended spin dynamics and correlation properties of a random magnetic field that is characterized by two independent parameters, its amplitude and correlation timescale, and which is considered generic and pertinent to a more realistic description of spin relaxation phenomena than the standard extreme narrowed approximation could afford. Section 3 then examines the detailed spectral and relaxation properties arising for an ensemble of spins immersed in such a field, including the spectral moments and relaxation times. We conclude in §4 with a discussion of the prospective implications and significance of the generalized spectral properties that we have introduced, in particular with regard to a potentially more fine-grained scheme for characterization and classification that is facilitated by the generalized spectral model.

## 2. Spin dynamics

A treatment that incorporates more sophisticated and realistic aspects of the random field would normally need to be perturbative in practice to facilitate tractability, whereas one that is exact would place unrealistic restrictions on that field; it is therefore gratifying that the present analysis treats a temporally correlated field in an exact *non*-perturbative manner, and thereby it is believed that the results provided herein may have particular significance in relation to the established literature and analytical spectral methods employed in spin relaxation, which date back to the seminal work of Lorentz [15].

We recall a pertinent theorem of Doob [16], which states that a process that is Gaussian, stationary and Markov is realized by a linear SDE, and is therefore of Ornstein–Uhlenbeck type. With regard to the domain of validity of such a trio of stochastic assumptions: for the first, one may consider the random field as arising as an aggregate of a fixed large number of independent fields, as would be envisioned in a multi-molecular environment. For stationarity, one may consider a time window of interest, consisting of many field correlation times, over which the statistical character of the process remains approximately the same, so that any non-stationary behaviour could be accounted for separately as a broad timescale modulation type effect (cf. the treatment of non-Gaussian scattering in [17–19]). The Markov property is more difficult to justify in general, and has the status here of a valid working assumption at the level of the driving random magnetic fields (as distinct from the ensuing spin process). In this respect, it is considered reasonable that any ‘memory’ effect possessed by these fields, whereby the future state is dependent on its previous history in addition to the current state, is rendered negligible by comparison to the effect of instantaneous interactions through neighbouring fields. In other words, the current state of the random fields is, to all intents and purposes, a sufficient statistical descriptor for all subsequent evolution. In what follows, nonetheless, we will still allow for the property of temporal correlation in the random field, which indeed is the feature that extends our analysis beyond extreme narrowing.

To avoid any ambiguity with regard to the Stratanovich versus Ito calculus—it is the *Ito* form that is applied here and in earlier cited work by the author.

### 2.1. Extended spin dynamics

In general [1], the perturbing random magnetic field is described via an isotropic temporally *correlated* (three-dimensional) Ornstein–Uhlenbeck process $h_{t}$ with associated random Hamiltonian:

(2.1) $$\begin{array}{rcl} H_{1}(t) & = & \frac{1}{2}\sigma \cdot \left(h_{t}^{\left(x\right)},h_{t}^{\left(y\right)},h_{t}^{\left(z\right)}\right)\\ & = & \frac{1}{2}\left(\begin{array}{cc} h_{t}^{\left(z\right)} & h_{t}^{\left(x\right)}-ih_{t}^{\left(y\right)}\\ h_{t}^{\left(x\right)}+ih_{t}^{\left(y\right)} & -h_{t}^{\left(z\right)} \end{array}\right), \end{array}$$

in which σ is the standard three-vector of Pauli matrices. In the analysis that follows, we shall restrict our attention to axial (longitudinal) field fluctuations, i.e. pure de-phasing, since this is more tractable and retains the essential features of the correlated random field model that we wish to capture. The equations of motion for the (pure state) density matrix M in the interaction representation are then

(2.2) $$\frac{1}{i}\frac{\text{d}M^{*}}{\text{d}t}=-[H_{1}(t),M{]}^{*},$$

in which the perturbation $H_{1}(t)$ is a stationary operator-valued random process, and equation (2.2) is expressed according to the interaction picture convention that

(2.3) $${\hat{O}}^{*}(t):=e^{iH_{0}t}\hat{O}e^{-iH_{0}t},$$

for an arbitrary observable operator $\hat{O}$, and in which $H_{0}$ is the background Hamiltonian (arising from the main magnetic field).

### 2.2. Correlated random fields

Accordingly, we take the random field to follow the Ornstein–Uhlenbeck SDE

(2.4) $$\text{d}h_{t}=-\frac{1}{2}\beta h_{t}\text{d}t+\kappa \beta^{1/2}\text{d}W_{t},$$

which consequently has an autocorrelation time 1/β and asymptotic variance $\kappa^{2}$. (Note that the appearance of κ here can be seen to arise through the scaling $h=\kappa h^{\left(n\right)}$, where $h^{\left(n\right)}$ is the normed-variance process obtained from equation (2.4) by setting κ=1.) Thus, the quantity κ represents the amplitude of the random magnetic field. Observe here that the constants β and κ both have dimensions of frequency, the Wiener differential dW has dimensions of square-root time, and we have adopted natural units with the reduced Planck’s constant ℏ=1 (so that the unit of action, ‘energy × time’, is dimensionless and the Hamiltonian is considered to have dimensions of frequency). It is a straightforward matter to restore Planck’s constant on dimensional grounds at any stage of these calculations.

It should also be observed, with respect to the equation for the phase differential and its dependence on the random field amplitude scaling, the correspondence between the current treatment (as represented here by equation (2.4)) and the extremely narrowed approach of [1] (specifically the constant k of eqn (32) therein). In the latter, there is only that single parameter (cf. our later discussion on the narrowing limit herein, where β→∞), and in comparison both constants k and κ have dimensions of frequency. (Observe in this regard that the white noise process in [1], through its δ-function autocorrelation, has dimensions of square-root frequency, which establishes dimensional consistency.)

Now, the solution of equation (2.4) is explicit and well known—see, for example, §8.1 of [17] for an accessible exposition of the (complex) Ornstein–Uhlenbeck equation. The phase dynamics in this case can thus be obtained via an exact integration of this SDE for the driving random field. (We provide the complete mathematical details of the key derivations that follow in the remainder of this section in appendix A.) Accordingly, we find the incremental transverse phase process $\Delta \phi (t,t^{\prime }):=\phi_{t}-\phi_{t^{\prime }}$ satisfies

(2.5) $$\Delta \phi =\omega_{0}(t-t^{\prime })-\frac{2}{\beta }\left\{h_{t}-h_{t^{\prime }}-(\kappa^{2}\beta {)}^{1/2}(W_{t}-W_{t^{\prime }})\right\},$$

and the resulting spin/phase processes are non-Markov. The spin autocorrelation function, via the MSD, amounts to the characteristic function of the incremental phase. Since the latter is Gaussian, we find

(2.6) $$\left\langle s_{t}s_{t^{\prime }}^{*}\right\rangle =\exp\left(-\frac{1}{2}\mathrm{Var}(\Delta \phi )\right).$$

Now, the variance term can be computed from the above solution (2.5) for the phase increment. Thereby, we find the following explicit *exact* form for the autocorrelation:

(2.7) $$R_{S}(\tau )=\exp\left(-\frac{2\kappa^{2}}{\beta }|\tau |\right)\times \exp\left\{-\frac{4\kappa^{2}}{\beta^{2}}\left(-1+\exp\left(-\frac{1}{2}\beta |\tau |\right)\right)\right\},$$

wherein, of special significance, we notice the appearance of the (negative) *double-exponential* exp⁡(exp⁡(⋅)) in the second factor. Indeed, this feature extends our *real-time* analysis to a class of functions that are not part of the mainstream in the contemporary spin relaxation (NMR/EPR) literature or methodologies, which have spectral origins dating back to Lorentz [15]. These independent derivations we have presented here, developed via the MSD and stochastic calculus as an extension of the dynamical theory of spin noise and relaxation originated in [1] to describe the resonance as a real-time process for an ensemble of spins, should be compared to the existing theory of stochastic line broadening developed originally in [20–23]. The latter works are based on a general form posited for the autocorrelation function of the random frequency fluctuations, and thereby incorporate the form (2.7) as a special case. This specific form for the autocorrelation and its associated power spectral density (PSD), alongside the various consequences in our fully dynamical framework that we shall describe in the sections that follow, *most notably the real-time dynamical derivation, the spectral-moment analysis and the parameter-extraction scheme*, constitute our key results here. The effect of the temporally correlated random field is to render the spin process non-Markovian, as follows immediately from Doob’s theorem. The spin process is nevertheless ergodic owing to sufficiently fast decay of the autocorrelation. Specifically, the mean-square ergodic property is established via a condition on the spin auto-covariance function—see appendix B.

Consistently, the autocorrelation function is broadened, and the corresponding PSD is narrowed. (With regard to a putative ‘closed form’ expression for the latter, it appears that the Fourier transformation entailed here does *not* result in a mathematical expression that is expressible in terms of existing ‘known functions’, and thereby our expression of the spectral density, which is mathematically exact, remains here in implicit integral form. Of course, as in other contexts, one could introduce a new ‘special function’ into the mathematical dictionary to accommodate the analysis here, and then our spectral result would have a compact appearance in terms of this new function, whose properties can be determined and documented by studying the ancestor integral.) Naturally, for ‘low’ frequencies (i.e. close to Larmor), the spectrum appears close to Lorentzian, but for high frequencies the difference is more apparent, with the tails of the new spectrum being narrower. Indeed, the asymptotic behaviour of the new spectrum compared to Lorentzian is functionally quite different, which feature is exemplified by considering the existence, or otherwise, of spectral moments, as we shall do presently.

## 3. Spectral and relaxation properties

Here, we explore the detailed spectral consequences of the correlated random field model introduced above, in terms of spectral moments and the level crossing rate (LCR) familiar from other areas, including communications signal processing [24]. In addition to providing some simulations of the random field and ensuing phase/phasor behaviour, we discuss the interpretation of corresponding notions of relaxation times that differ in significant respects from those applied as standard.

### 3.1. Spectral moments and level crossing rate

The frequency moments of a PSD or ‘spectral moments’ are important tools in interpreting resonance spectra, and enable classification of different types of chemical environments through relaxation behaviour. In what follows, we shall see that these spectral features are quite different for the correlated models considered here, as compared to the standard type (Lorentzian) models.

The LCR measures the infinitesimal rate of crossings of a stochastic process above and below some given threshold, and is relevant in applications of stochastic process theory generally, and also in the theory of communications channels, for example. An analysis in this respect equates such rate to the second moment of the PSD, and thereby the second derivative of the autocorrelation function, at time lag zero [24].

The cases under investigation here show some interesting features in these regards. Firstly, in the case of extreme narrowing where the spectrum is Lorentzian, it is familiar that the second-order spectral moment is positive infinite, and correspondingly the (first and) second derivative(s) of the autocorrelation function (do) does not exist. Intuitively, this is consistent with the observation that a sample path of the spin process, essentially Ornstein–Uhlenbeck, appears ‘jagged’ in character—in precise mathematical terms it is continuous/non-differentiable everywhere—a feature that can be appreciated in terms of the scaling of the Wiener (phase) differential with the *square root* of the infinitesimal time increment. Now, closer scrutiny of equation (2.7), setting $\alpha =\kappa^{2}/\beta$ (which therefore also has dimensions of frequency), reveals that the autocorrelation, a symmetric function, is controlled by the two parameters α,β according to

(3.1) $$R(\tau )=e^{4\alpha /\beta }\exp\left\{-2\alpha |\tau |-\frac{4\alpha }{\beta }\exp\left(-\frac{1}{2}\beta |\tau |\right)\right\}.$$

Thereupon, expanding in powers of the (positive or negative) lag time around τ=0, we find the entirety of the exponent within the brace immediately above to be

(3.2) $$-\frac{4\alpha }{\beta }\left(1+\frac{1}{8}\beta^{2}\tau^{2}-\frac{1}{48}\beta^{3}|\tau {|}^{3}+o(\tau^{3})\right),$$

in which terms linear in τ have cancelled. Putting this together, we find the autocorrelation in equation (3.1) becomes

(3.3) $$\begin{array}{c} R(\tau )=\exp\left(-\frac{\alpha \beta }{2}\tau^{2}+\frac{\alpha \beta^{2}}{12}|\tau {|}^{3}+o(\tau^{3})\right)\\ =1-\frac{\alpha \beta }{2}\tau^{2}+\frac{\alpha \beta^{2}}{12}|\tau {|}^{3}+o(\tau^{3}). \end{array}$$

Therefore, the second derivative exists and is negative, $\frac{{\text{d}}^{2}R}{\text{d}\tau^{2}}{|}_{0}<0$; for the third derivative, $\frac{{\text{d}}^{3}R}{\text{d}\tau^{3}}{|}_{0^{+}}=-\frac{{\text{d}}^{3}R}{\text{d}\tau^{3}}{|}_{0^{-}}$, there is a simple discontinuity; consequently, the fourth and higher derivatives $\frac{{\text{d}}^{n}R}{\text{d}\tau^{n}}{|}_{0}$, n≥4 do not exist. Since the PSD S(ω) and autocorrelation R(τ) are a Fourier pair, $R(\tau )=\frac{1}{2\pi }\int e^{i\omega \tau }S(\omega )\;\text{d}\omega$, the spectral moments can be obtained as the zero-time derivatives of R:

(3.4) $$\left\langle (i\omega {)}^{n}\right\rangle ={2\pi \frac{{\text{d}}^{n}R}{\text{d}\tau^{n}}|}_{\tau =0}.$$

Thereby, for the spectral moments of our correlated random field model, the first is zero, without (unlike the Lorentzian case) any need for a Cauchy principal value. The second is *finite*, $\langle \omega^{2}\rangle <\infty$, and so the spin process has a finite LCR, as can be intuited from figure 1 for a random field and corresponding phase sample paths; this is illustrated as exponentiated or ‘phase wrapped’ in figure 2. The third spectral moment is ill-defined (unless a Cauchy principal value is taken, in which case it is zero); the fourth moment and all even moments of higher order are infinite, while higher-order odd moments are ill-defined as above.

**Figure 1.**
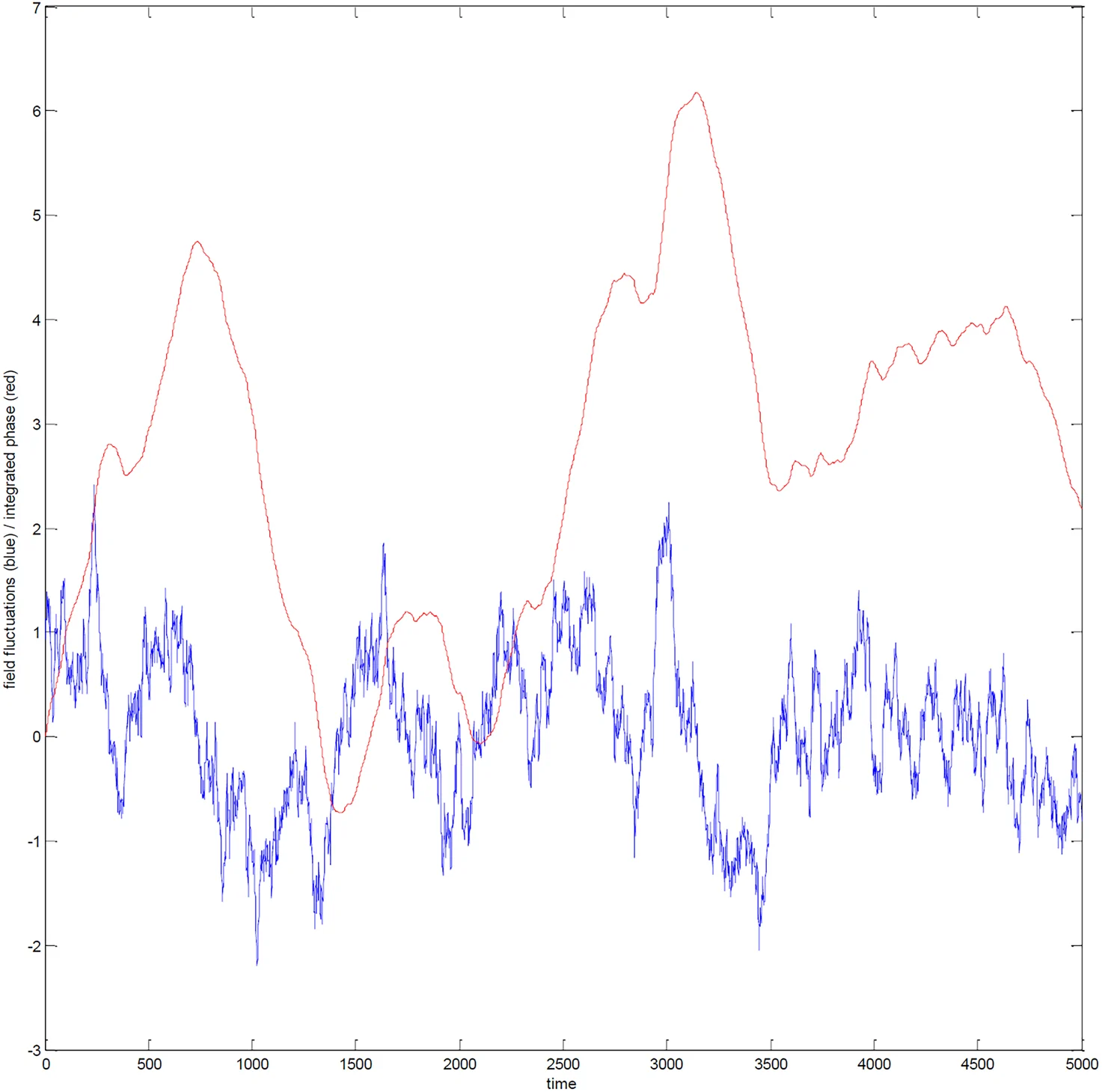
Time series overlay of random field (blue) and corresponding random phase (red)—observe memory in the phase process, whose derivative exists and is continuous (unlike the case of extreme narrowing). Parameter values: $\kappa =10^{-4}$, $\beta =10^{-3}$.

**Figure 2.**
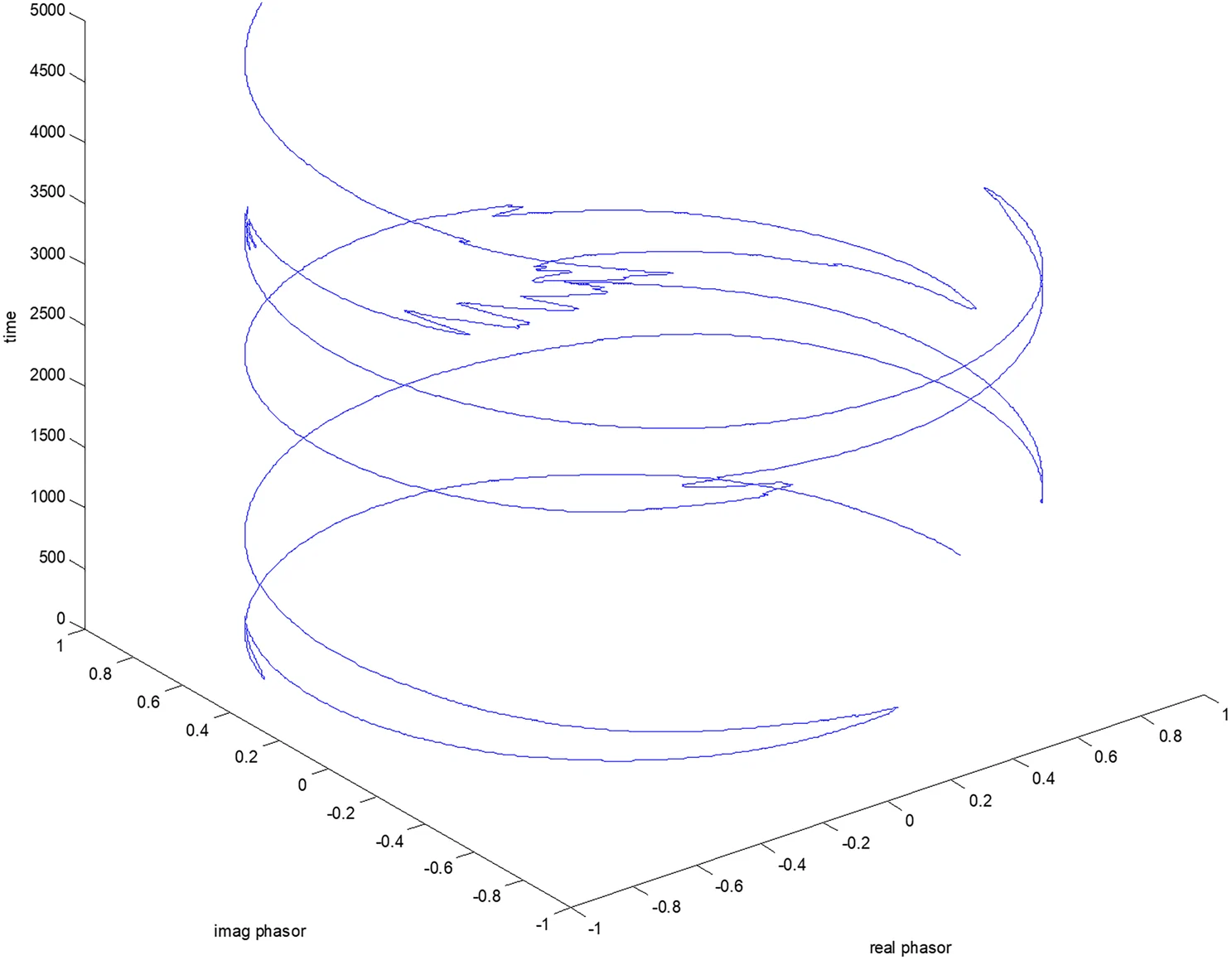
Random phasor trajectory for a single spin—observe the frequent changes in direction which occur at the zero crossings of the random field. Parameter values: $\kappa =10^{-4}$, $\beta =10^{-3}$ as in figure 1.

The situation is therefore quite unlike that of extreme narrowing, where the second spectral moment, and all higher-order even moments, are infinite, and the spin process has an *infinite* LCR. The latter description should therefore be viewed as an extreme mathematical idealization which is ultimately unphysical. The appeal of our more general description in these respects is thereby evident, from both a mathematical and experimental point of view. It should be emphasized that the more general approach taken here does not merely pertain to the appropriate choice or range of suitable parameters, as may be pertinent to specific scenarios, chemical environments and so on, but instead effects an essential departure from the class of Lorentzian spectra that are traditionally employed. The new class of spectra could be of considerable significance in terms of matching to real experimental data, for example. As discussed, the differences are more apparent away from the modal frequency or ‘off resonance’.

### 3.2. Interpretation and relaxation times

Our analysis rather crucially suggests the adoption of new and alternative measures of relaxation timescales, via incorporation of the second spectral moment, which is now finite (and indeed is only so *without* the extreme narrowed restriction). Thereby, the reciprocal of the spectral standard deviation (the latter defined as the square root of this second spectral moment), multiplied by 2π, gives an alternative additional natural timescale $\tau_{\mathrm{spect}}$. Incorporating the effects of the amplitude for a correlated random field fully could yield essential alternatives for spectroscopic calibration and classification purposes, and lies in comparison to the usual (reciprocal) ‘full-width-half-maximum’ (FWHM) that is employed (which is proportional to 1/k in equation (1.9)), and in the context of the present treatment pertains only to the *single* parameter α.

To demonstrate more explicitly this relationship between the general case and that of extreme narrowing, referring to equation (3.1), we can rewrite this, factoring suggestively, as

(3.5) $$R(\tau )=\exp(-2\alpha |\tau |)\times \exp\left(\frac{4\alpha }{\beta }\left(1-e^{-(1/2)\beta |\tau |}\right)\right).$$

Notice that the second factor increases from unity at τ=0 to an asymptote $e^{4\alpha /\beta }>1$, which effectively broadens the autocorrelation relative to the extreme narrowed case. The latter is represented by the first factor *alone*, and obtained via the limit β→∞, which renders the second factor equal to a constant of unity. This is well illustrated in figure 3, which depicts the behaviour of equation (3.5) for fixed α and varying β. (For varying values of α, this would affect the sharpness of the non-differential apex of the limiting curve for infinite β, having modulus gradient equal to 2α.) Explicitly, from equations (3.3) or (3.5) and (3.4), the second moment of the spectral density is

(3.6) $$\left\langle \omega^{2}\right\rangle =-2\pi {\text{d}}^{2}R/\text{d}\tau^{2}{|}_{\tau =0}=2\pi \alpha \beta .$$

In turn, we see from the way that α was introduced above (3.1) in terms of κ (namely $\alpha :=\kappa^{2}/\beta$), that this leads to a new spectral timescale $\tau_{\mathrm{spect}}$ via the identification through the quadratic moment

(3.7) $$\tau_{\mathrm{spect}}:=2\pi {\left\langle \omega^{2}\right\rangle }^{-1/2}=\sqrt{\frac{2\pi }{\alpha \beta }}=\frac{\sqrt{2\pi }}{\kappa }.$$

This quantity, which arises only in the presence of field correlations, is quite distinct from the ordinary notion of relaxation time, and relates to the amplitude of the driving random field, as represented by κ in equation (2.4), in a very natural way. Notice here that, whereas $\tau_{\mathrm{spect}}$ depends on both frequency parameters α and β, it only depends on the single parameter κ through the product αβ. Moreover, this is evident from the $O(\tau^{2})$ term in equation (3.3), which reveals the second time derivative (at zero lag) to depend only on this product. (Notice that higher-order terms in this expansion do not possess this property.) Such preservation of the spin process second spectral moment under temporal re-scalings of the driving random field constitutes an interesting result in itself. It can also be interpreted from a functional (Fourier) point of view, since the moment $\left\langle \omega^{2}\rangle =\int \omega^{2}S(\omega \right)$ is *global* in frequency but *local* in (a neighbourhood of zero lag) time, thus (as we have explained in the derivations earlier) proportional to ${\text{d}}^{2}R/\text{d}\tau^{2}{|}_{0}$. Although the *global* shape of the autocorrelation function depends on β, the (zero lag) second time derivative (which, since the first derivative vanishes (⟨ω⟩=0), equates to the local curvature of the autocorrelation function), does not. As an illustration of this situation, for example, we could consider, for fixed κ, a case of ‘extreme broadening’ represented by the limit β→0 which (recalling $\alpha \beta =\kappa^{2}$) also entails α→∞. Again, from equation (3.3), we can write $R=\exp\left\{\kappa^{2}\left(-\frac{1}{2}\tau^{2}+\frac{1}{12}\beta \tau^{3}+o(\beta )\right)\right\}$ and so this limit yields an asymptotic *Gaussian* autocorrelation:

(3.8) $$R_{\mathrm{Gauss}}(\tau )=\exp\left(-\frac{1}{2}\kappa^{2}\tau^{2}\right),$$

and the corresponding spectral density is therefore also Gaussian:

(3.9) $$S_{\mathrm{Gauss}}(\omega )=\frac{\sqrt{2\pi }}{\kappa }\exp\left(-\frac{\omega^{2}}{2\kappa^{2}}\right).$$

In such a case, it is particularly striking to observe, since α→∞, that the standard relaxation time T2→0. Nonetheless, equation (3.8) presents a natural timescale. (As an alternative example, we can also consider the limit β→0 with α fixed, so that the variable amplitude parameter κ→0. This is illustrated by figure 3, as the broadening sequence of curves. Then once again from equation (3.3), the exponent therein tends to zero and so the spin autocorrelation tends to a constant function. Accordingly, the spin PSD is a zero-frequency delta-function, and $\tau_{\mathrm{spect}}$ is infinite.)

**Figure 3.**
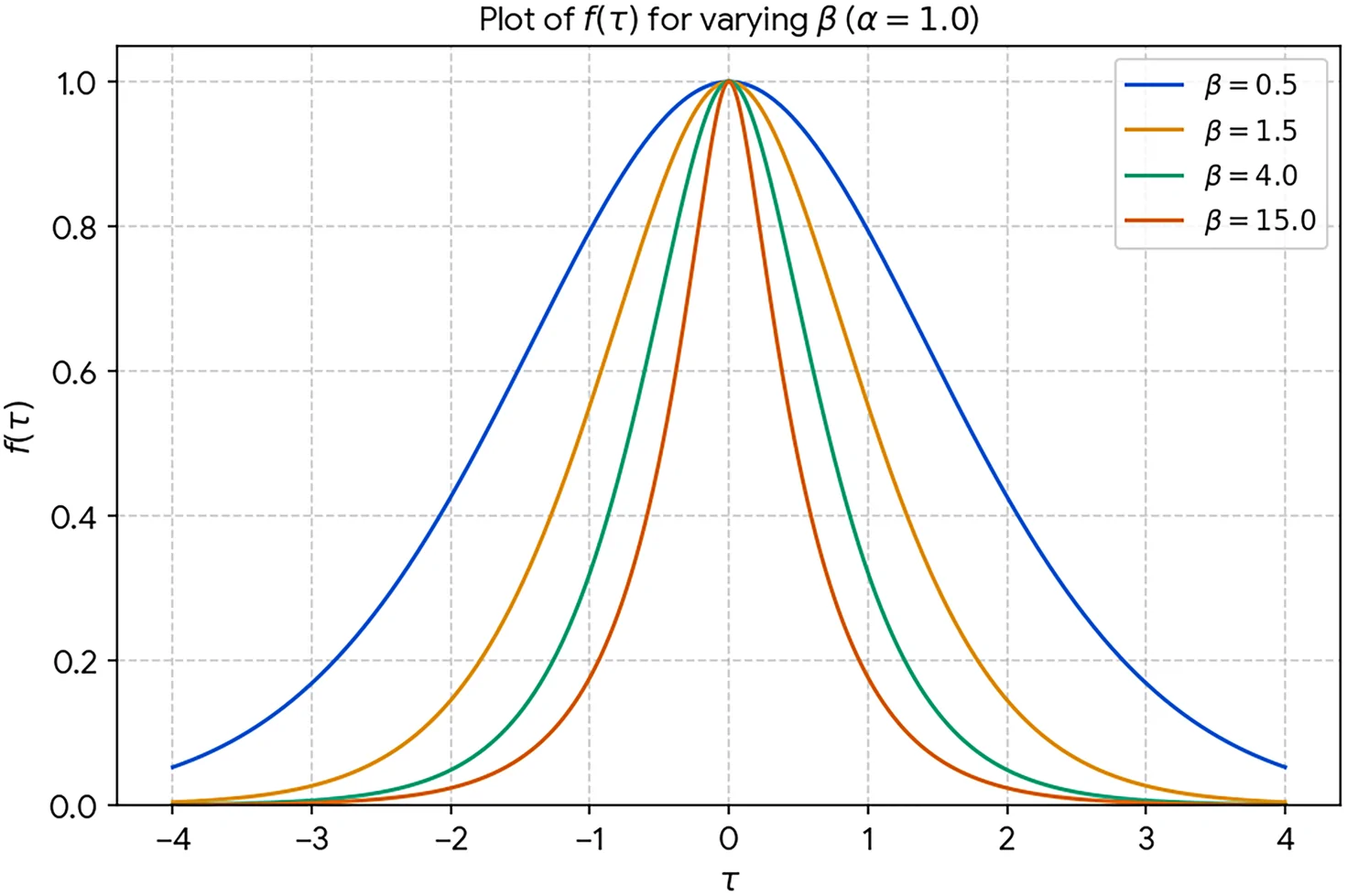
Illustration of the broadening of the spin autocorrelation function (3.5) as the random field temporal correlation parameter β introduced in equation (2.4)—conversely as β→∞ extreme narrowing is approached, and the function approaches a (symmetric) pure exponential decay that is characteristic of the Lorentzian spectrum (and ultimately unphysical). (For varying values of α, this would affect the sharpness of the non-differentiable apex of the limiting curve for infinite β, having modulus gradient equal to 2α.).

Some further detailed discussion of the asymptotics and the delicate interplay between the various parameters involved here is provided in appendix C.

## 4. Summary and prospects

In this article, we have investigated the behaviour of an ensemble of spins immersed in a random magnetic field that is temporally correlated in accordance with a set of generic statistical assumptions, thus extending beyond the extreme narrowed approximation. We have derived the resulting stochastic spin dynamics and ensuing spectral properties, and contrasted these with the standard results. Our treatment incorporates both the amplitude and correlation time/spectral width of the driving random field, as two independent parameters. The spin autocorrelation function emerges in a double-exponential form, with a broadened autocorrelation/narrowed spectrum compared to the Lorentzian case. Recovery of extreme narrowing is obtained as a certain limit (β→∞), yielding a Lorentzian spectrum—moreover, the spectrum is *only* Lorentzian in this limit. It should be emphasized that this analysis has been *non-*perturbative in the random field.

Our discussion more broadly points towards a substantial shift in point of view, for spectroscopic calibration and classification purposes, away from a single ‘relaxation time’ parameter, towards the spin correlation function *itself*, and its associated spectral density, which, as we have seen, depends on *two* independent parameters. While it is appreciated, as pointed out in §1, that data-driven analyses that exploit the entirety of the spin autocorrelation function have been studied previously, the present results offer a legitimate and principled alternative in the form of a generic *finite* parametric family of spectra/autocorrelation functions that are accordingly applicable to a wide range of applications.

In such a new paradigm, it is essential that the spectral model be the mathematically *correct* and more general *non*-Lorentzian one. In this respect, we envisage adoption of a two-parameter data-fitted model according to equation (3.5), incorporating both the Lorentzian and spectral narrowing contributions arising from field correlation, which would be an improvement on the current state of the art. In terms of classification, there are well-established measures of (dis-)similarity for spectra that could be employed, such as the familiar statistical distance in Hilbert space [25]. In the present context, this would have implications for any application that involves (T2-weighted) imaging, for which there is a vast literature. An image in this framework would be not just a measure of T2 (variants), as it is (they are) distributed over space, but a more refined map of how the detailed spectral properties vary—for example, an image could be generated by plotting for each spatially resolved cell an intensity that is determined by the statistical distance of the PSD at that point from that of some chosen reference point within the image. Such a proposal incorporates more sophisticated information about the physical behaviour of the spins, and would presumably therefore lead to more discriminating ability in the images thus generated.

The dynamical modifications entailed here also have direct implications for the further study of real-time properties [3], and will be described separately. In this regard, we discuss in some further detail the question of parameter extraction in appendix D. An extension to higher spins, specifically a system of entangled spin-1/2 particles, was tackled in [4], albeit in the extreme narrowed limit—we expect a corresponding analysis would go through for the present non-extreme narrowed case, and this will be explored in later work.

## Supplementary Material



## Data Availability

The present article contains two figures based on simulated data—these data and associated code used to generate them are freely available as supplementary material [26].

## Appendix A. Spin-phase dynamics

In this appendix, we provide the complete mathematical details surrounding the development of §2.2, specifically equations (2.5) and (2.6), leading to the central result (2.7). We begin by expositing the (exact stochastic) solution to the random field SDE (2.4), which we recall as

(A 1) $$\text{d}h_{t}=-\frac{1}{2}\beta h_{t}\text{d}t+\kappa \beta^{1/2}\text{d}W_{t}.$$

By expanding the differential $\text{d}\left(e^{\frac{1}{2}\beta t}h_{t}\right)=e^{\frac{1}{2}\beta t}\left(\text{d}h_{t}+\frac{1}{2}\beta h_{t}\text{d}t\right)$ and equating the factor in parentheses on the right-hand side to $\kappa \beta^{1/2}\text{d}W_{t}$ via equation (2.4), we find via an integration $\int_{0}^{t}$

(A 2) $$\int_{0}^{t}\text{d}\left(e^{\frac{1}{2}\beta s}h_{s}\right)=\kappa \beta^{1/2}\int_{0}^{t}e^{\frac{1}{2}\beta s}\text{d}W_{s}.$$

Thereby, we find the solution

(A 3) $$h_{t}=e^{-\frac{1}{2}\beta t}h_{0}+\kappa \beta^{1/2}\int_{0}^{t}e^{\frac{1}{2}\beta (s-t)}\text{d}W_{s}.$$

Likewise, and more amenably in what follows here, applying the same argument with $\int_{t^{\prime }}^{t}$ (for $t^{\prime }<t$) yields

(A 4) $$h_{t}=e^{-\frac{1}{2}\beta (t-t^{\prime })}h_{t^{\prime }}+\kappa \beta^{1/2}\int_{t^{\prime }}^{t}e^{\frac{1}{2}\beta (s-t)}\text{d}W_{s}.$$

To calculate the effect of the random field on the phase process, consider equation (2.2), which for longitudinal (axial) field fluctuations yields the phase differential (§IIIC in [1])

(A 5) $$\text{d}\phi_{t}=h_{t}\text{d}t.$$

(The analysis for isotropic field fluctuations is more complicated and treated in the extreme narrowed approximation in [1].) Combining this with equation (2.4), we deduce

(A 6) $$\text{d}\phi_{t}=-\frac{2}{\beta }\left(\text{d}h_{t}-\kappa \beta^{1/2}\text{d}W_{t}\right),$$

leading directly to the incremental transverse phase (2.5) which (for $\omega_{0}=0$ or ‘on-resonance’) becomes

(A 7) $$\Delta \phi =-\frac{2}{\beta }\left\{h_{t}-h_{t^{\prime }}-(\kappa^{2}\beta {)}^{1/2}(W_{t}-W_{t^{\prime }})\right\}.$$

Equipped with these various expressions, we can now explicitly compute the spin-phasor autocorrelation function. For an MSD defined by

(A 8) $$\Sigma_{t}:=\frac{1}{N^{1/2}}\sum s_{t}^{\left(j\right)},$$

in which the constituent phasor is $s_{t}^{\cdot }=\exp(i\phi_{t}^{\cdot })$ (spin label j), with its autocorrelation function $\left\langle s_{t}s_{t^{\prime }}^{*}\right\rangle$, we find equivalence of the MSD and spin-phasor autocorrelation functions. This arises through the equality of expectations

(A 9) $$\left\langle \Sigma_{t}\Sigma_{t^{\prime }}^{*}\right\rangle =\left\langle s_{t}s_{t^{\prime }}^{*}\right\rangle ,$$

which follows from the phasors (spins) being mutually independent. Now, we observe that according to equation (A 5), the phase process $\phi_{t}$ is a Gaussian random process (GRP) since the random field process $h_{t}$ has this property (it is an Ornstein–Uhlenbeck process). (A general property of GRPs is their preservation under linearity and limits.) To see these properties explicitly via stochastic integration, and usefully for our calculations here, the solutions (A 3), (A 4) are manifestly Gaussian. For the phase increment, Gaussianity follows explicitly from the properties of stochastic integrals by writing (A 7) as

(A 10) $$\Delta \phi (t,t^{\prime })=-\frac{2}{\beta }\left\{\left(e^{\frac{1}{2}\beta (t^{\prime }-t)}-1\right)h_{t^{\prime }}+\kappa \beta^{1/2}\int_{t^{\prime }}^{t}\left(e^{\frac{1}{2}\beta (s-t)}-1\right)\text{d}W_{s}\right\}.$$

Now, the above expression, in addition to manifesting the Gaussian property, provides a convenient way to calculate the first and second moments of the incremental phase, which characterize it completely. Clearly, ⟨Δϕ⟩=0, and so $\mathrm{Var}[\Delta \phi ]=\langle (\Delta \phi {)}^{2}\rangle$. For the latter, setting the lag time $\tau :=t-t^{\prime }$ (for $t>t^{\prime }$, so τ>0),

(A 11) $$\begin{array}{rl} \left\langle {\left(\Delta \phi \right)}^{2}\right\rangle & =\frac{4}{\beta^{2}}\left\langle {\left(e^{-\frac{1}{2}\beta \tau }-1\right)}^{2}h_{t^{\prime }}^{2}\right\rangle \\ & \;+\frac{4\kappa^{2}}{\beta }\left\langle \left[\left(\int_{t^{\prime }}^{t}(e^{\frac{1}{2}\beta (s-t)}-1)\text{d}W_{s}\right)\left(\int_{t^{\prime }}^{t}(e^{\frac{1}{2}\beta (s^{\prime }-t)}-1)\text{d}W_{s^{\prime }}\right)\right]\right\rangle , \end{array}$$

wherein cross terms arising from the square of equation (A 10) involving $h_{\cdot }\;\text{d}W_{\cdot }$ vanish under the expectation. On the right-hand side above, for the first term, we recall from the discussion surrounding (2.4) that $\langle h^{2}\rangle =\kappa^{2}$. To tackle the second term, we require the *Ito isometry* (see, for example, §2.1.1 in [17] for an accessible approach or [27]), which has its origins in the independent increments property and finite quadratic variation defining properties of Brownian motion (synonymously the Wiener process), and which equates this expectation of a product of stochastic integrals to an (ordinary) integral of an expectation, according to the rule $E\left[\left(\int_{t^{\prime }}^{t}\phi_{s}\text{d}W_{s}\right)\left(\int_{t^{\prime }}^{t}\psi_{s^{\prime }}\text{d}W_{s^{\prime }}\right)\right]=\int_{t^{\prime }}^{t}E\left[\phi_{s}\psi_{s}\right]\text{d}s$. Applying this rule to the expectation in the second term (without the constant pre-factor), yields the expression

(A 12) $$\int_{t^{\prime }}^{t}{\left(e^{\frac{1}{2}\beta (s-t)}-1\right)}^{2}\text{d}s,$$

which, on direct integration, becomes

(A 13) $$\tau +\frac{1}{\beta }\left(-3+4e^{-\frac{1}{2}\beta \tau }-e^{-\beta \tau }\right).$$

Now, combining the various parts from equation (A 11), we find, upon substantial simplification among the terms involved, the somewhat more compact and elegant relation for the incremental phase variance

(A 14) $$\left\langle {\left(\Delta \phi \right)}^{2}\right\rangle =\frac{4\kappa^{2}}{\beta^{2}}\left(\beta \tau +2e^{-\frac{1}{2}\beta \tau }-2\right).$$

Now, the spin-phasor autocorrelation (A 9) amounts to the characteristic function of the incremental phase $\left\langle s_{t}s_{t^{\prime }}^{*}\rangle =\langle \exp i\Delta \phi \right\rangle$. Since that increment is Gaussian, as explained above, this assumes a particularly simple form

(A 15) $$\left\langle s_{t}s_{t^{\prime }}^{*}\right\rangle =\exp\left(-\frac{1}{2}\mathrm{Var}[\Delta \phi ]\right),$$

which, on substituting equation (A 14), yields the claimed relation (2.7). (In doing so here in equation (A 15), we have exploited the characteristic function of a normal variate $N∼N(\mu ,\sigma^{2})$, which takes the convenient and simple form $\Phi_{N}(\omega ):=E\left[e^{i\omega N}\right]=e^{i\mu \omega -\frac{1}{2}\sigma^{2}\omega^{2}}$, whereupon setting ω=1 yields the required relation.)

## Appendix B. Ergodicity

In this appendix, we examine the ergodic property of the spin-phasor process. The non-Markovian spin process discussed in §2.2 is nevertheless ergodic owing to the sufficiently fast decay of its autocorrelation. Specifically, the mean-square ergodic property—that the time-window average tends to the stationary ensemble average in the sense of mean-square convergence as the size of the time window tends to infinity—is established via a condition on the auto-covariance function, as follows. For a time window moving averaged process $Y_{t}:=\frac{1}{2T}\int_{t-T}^{t+T}X_{s}\text{d}s$ (which is stationary if X is), the auto-covariance functions of X and Y, denoted respectively as $C_{X}$ and $C_{Y}$, can be shown to be related via the integral expression (see ch. 12 in [28]) $C_{Y}(\tau )\equiv \frac{1}{2T}\int_{-2T}^{2T}C_{X}(\tau -s)\left(1-\frac{\left|s\right|}{2T}\right)\text{d}s$. Defining $\eta_{T}:=\frac{1}{2T}\int_{-T}^{T}X_{s}\text{d}s$, which is random and an unbiased estimator of η=E[X], and $\sigma_{T}^{2}=\mathrm{Var}[\eta_{T}]$, the process $X_{t}$ is said to be *mean-ergodic* if and only if $\sigma_{T}^{2}\to 0$ as T→∞—in other words, the windowed time average $\eta_{T}$ tends to its statistical expectation in the mean square sense as the size of the time window approaches infinity, and so in this sense the time average synthesizes the ensemble average. Setting τ=0, $\sigma_{T}^{2}=C_{Y}(0)$, which substituting into the above identity relating the two auto-covariance functions, yields a necessary and sufficient condition for X to be mean-square ergodic,

(B 1) $$\frac{1}{T}\int_{0}^{2T}C_{X}(s)\left(1-\frac{s}{2T}\right)\text{d}s\to 0,$$

as T→∞. Now, considering the specific form for the autocorrelation function of the spin-phasor process (2.7), it is evident that the required condition for ergodicity (B 1) is satisfied (for example, by upper-bounding the second factor in equation (2.7) by the constant $e^{4\kappa^{2}/\beta^{2}}$).

## Appendix C. Asymptotics

In this appendix, we explore in further detail the asymptotic spectral and timescale properties of the spin process, somewhat exhaustively for the various mathematical cases that can arise, and provide a physical interpretation for each. We also indicate some ideas of the numerical values of dimensional quantities that would be relevant in real experimental situations.

### C.1. Extreme narrowing

First of all, it should be appreciated that, in the context of the parameters introduced in §§2 and 3, the extreme narrowed approximation arises for α fixed and the limit β→∞, κ→∞, in which we recall the product relation $\alpha \beta =\kappa^{2}$. In this case, a characteristic frequency, if equated to the second spectral moment is *infinite*, as described in the discussion surrounding (3.7). Attendantly, an attempt at a notion of characteristic timescale from this point of view renders it *zero*. On the other hand, we are familiar in standard treatments [2] with an alternative finite measure of timescale and spectral width, in the extreme narrowed approximation. Regarding the first factor in equation (3.5), we observe $F\left[e^{-2\alpha |t|}\right]=\frac{4\alpha }{4\alpha^{2}+\omega^{2}}$, where F denotes the Fourier transformation according to the standard convention. This has a half-maximum spectral power of $S_{\mathrm{HM}}=1/2\alpha$, which is attained at a half-maximum frequency of $\omega_{\mathrm{HM}}=2\alpha$, and thereby the FWHM frequency is $2\omega_{\mathrm{HM}}=4\alpha$. The so-called ‘spin–spin’ relaxation time, also referred to as T2, is the characteristic decay constant in equation (3.5), and thereby $T2=1/2\alpha =1/\omega_{\mathrm{HM}}$. In such approximation, this value appears to be the *only* candidate for a relaxation timescale.

### C.2. Non-extreme narrowing

There are several interesting cases to explore here, some more physically relevant than others, and the situation highlights the degree of non-uniqueness or arbitrariness in assigning a notion of relaxation timescale in this more general framework of a correlated random field. Indeed, as we discussed in §4, this perspective suggests a shift in viewpoint towards using the spin spectrum itself, as opposed to any single notion of timescale that comes alongside an inevitable arbitrariness, as the most appropriate choice.

#### C.2.1. Putative timescales

There exist (at least) three candidate timescales associated with the relaxation process that suggest themselves naturally from our formalism.

Case ①: we can define a quantity $\tau^{①}:=1/2\alpha$, which is simply the exponential decay constant of equation (3.5).

Case ②: considering again equation (3.5), define $\tau^{②}:=1/\omega_{\mathrm{HM}}$ in which $\omega_{\mathrm{HM}}$ pertains to the *entirety* of the expression for the spin autocorrelation; from the temporal broadening aspect of the correlated field, this quantity therefore *exceeds* the extreme narrowed limit, so $\tau^{②}>1/2\alpha$.

Case ③: as we have already discussed in §3.2 and presented in equation (3.7), there is a third intrinsic and fundamental physical timescale in the problem $\tau_{\mathrm{spect}}$ (that is not apparent in the extreme narrowed approximation), namely $\tau^{③}:=\frac{\sqrt{2\pi }}{\kappa }$.

Each of the quantities ⓘ presents itself as a legitimate timescale associated with the spin relaxation process in our formalism, for relaxation in the presence of correlated random fields. Now, in summary, it is instructive to compare these three timescales in the extreme narrowed limit β→∞ (α fixed). Case ① yields a timescale $\tau^{①}$ that is independent of β, and thereby neglects the effects of a correlated random field. In case ②, as β→∞ the second factor in equation (3.5) tends to the constant unit function and so $\tau^{②}\to \tau^{①}$ (with limit from above). Finally, for case ③, we have the intrinsic fundamental timescale $\tau^{③}\to 0$ and hence this important physical parameter is effectively discarded in the extreme narrowed approximation. To make this situation even more readily apparent in terms of what might be termed ‘relaxation timescales’, to illustrate the true dependence on the timescale $\tau_{\mathrm{spect}}=\tau^{③}$, expressing the pertinent parameters as $\beta =4\pi \tau^{①}/\tau^{③2}$ and $\alpha /\beta =\tau^{③2}/8\pi \tau^{①2}$, we can thus rewrite the entire spin autocorrelation function as

(C 1) $$R(\tau )=\exp\left(-\frac{\left|\tau \right|}{\tau^{①}}\right)\times \exp\left(\frac{\tau^{③2}}{2\pi \tau^{①2}}\left(1-e^{-2\pi ①|\tau |}/\tau^{③2}\right)\right).$$

Of less practical relevance, but still of interest and to complete the mathematical picture, are two cases that regard the (limiting) behaviour of the random field amplitude, introduced earlier in equation (2.4) as the parameter κ.

#### C.2.2. High-field limit

Firstly, from a field perspective, consider a high-field limit on a given fluctuation timescale, which thus arises from β fixed and κ→∞, and accordingly α→∞ (for fixed τ>0). In such case, writing the combined exponent of equation (3.5) as $\alpha \left[-2\tau +\frac{4}{\beta }\left(1-e^{-\frac{1}{2}\beta \tau }\right)\right]$ and recognizing that the factor in brackets in the latter expression is negative (since $1-\frac{\tau \beta }{2}<e^{-\frac{1}{2}\beta \tau }$, τ>0), we deduce in this limit that R→0 (for τ>0), which represents a situation of *instantaneous decoherence/relaxation*.

#### C.2.3. Fixed field amplitude

Secondly, if the random field amplitude κ is controlled, while its autocorrelation time tends to zero, so that (again from the product relation $\alpha \beta =\kappa^{2}$) we hold κ as constant, and β→∞, α→0 (which is unlike the regular extreme narrowed case explained above, where α would be constant), then the spin autocorrelation tends to a constant function, R→1 for all lag times τ. In this case, the spin phases and processes are constant in time, and the system is *static*.

The latter two observations are in accord with physical intuition—as the strength of field fluctuations increases, this drives relaxation faster; conversely, if the fluctuations are weak (which incidentally is the usual justification for perturbative treatments) then relaxation progresses slowly and the T2 time is ‘long’ (see appendix C.2.4 for typical values).

#### C.2.4. Experimental values

It is worth mentioning, though it is not a main focus of attention in the present article, some actual values of dimensional quantities that arise in typical laboratory scenarios, in order to make contact with prospective experimental research and, from that perspective, stimulate further interest in our mainly theoretical results. For neat liquid water, for example, α∼0.25, so T2∼3–5 s. For the parameter β, the fluctuating random magnetic field in liquid NMR experiments has autocorrelation time of the order of a picosecond, $10^{-12}\;s$, and therefore $\beta ∼10^{12}$. Now, from the familiar product relation $\alpha \beta =\kappa^{2}$, we find for the field amplitude parameter $\kappa^{2}∼0.25\times 10^{12}$, so that $\kappa ∼5\times 10^{5}$. This means from equation (3.7) that the intrinsic timescale that comes from the second moment of the spin spectrum is of the order of a microsecond, $\tau^{③}∼1\times 10^{-6}\;s$ (whereas it is zero for extreme narrowing). For the strength of the fluctuating Hamiltonian, restoring Planck’s constant ℏ (which for simplicity hitherto has been set equal to unity), we have $|H_{\mathrm{fluct}}|∼\hbar \kappa$, and so this perturbing energy interaction is of magnitude $|H_{\mathrm{fluct}}|∼5\times 10^{-29}\;J$.

Having made these parameter value identifications in a familiar experimental scenario, it should also be stressed that the parameters in our analysis are *free*, the treatment is exact and non-perturbative, and these features should be considered a strength of the current approach. It points towards a situation where these results develop rather broad experimental relevance.

We emphasize again that these sets of observations appear to be beneficial in terms of prospective principled alternatives to standard relaxation classification techniques, and at the same time resolve a fundamental pathology in the standard treatment where infinities arise, which is advantageous both conceptually and experimentally.

## Appendix D. Parameter extraction

We provide, in this appendix, a novel scheme for parameter extraction, namely the frequency parameters α, β and the field amplitude parameter κ introduced in the random field and ensuing spin processes described in §§2 and 3, for a *finite* value of β. Identification of these parameters, from either real or simulated data, may obviously be relevant to schemes for classification. Our purpose here is not to focus on the details of those types of schemes, of which there would be multitudes, but rather to indicate how to obtain the relevant parameter values from our formalism that would feed into such schemes as the essential variables. This question is not entirely trivial because, at face value, the expression (2.7), or the equivalent (3.1) and (3.5), do not suggest an immediate way to pull out these parameters from the function, unlike the extreme narrowed case where only the single parameter α is involved. Nevertheless, an expeditious procedure to achieve this is possible, and presents itself naturally in our current framework. (We do not claim this is the only scheme, but it is an interesting and natural one.)

Firstly, we have conveniently at our disposal the finite RMS frequency relations (3.6) and (3.7). Thereby, we have immediate access to the amplitude scale parameter κ from the spectral function or data, by calculating its second moment by direct quadrature. Next comes the issue of finding one of the two remaining parameters α,β, or an appropriate function involving at least one of them—once either of these parameters is determined, we then have the complete set, by virtue of the product relation $\alpha \beta =\kappa^{2}$. In order to proceed further in this regard, we go back to the random field amplitude equation (2.4), which for the ensemble acquires a spin label (j). According to the Ito calculus, it follows that the squared volatility of the individual field amplitude pertaining to spin (j) satisfies the finite quadratic variation relation $\text{d}h_{t}^{(j)2}=\kappa^{2}\beta \text{d}t$, which is independent of the spin label. Now, considering the MSD signal (A 8), its time derivative $P_{t}:=\text{d}\Sigma_{t}/\text{d}t$ (this derivative exists beyond extreme narrowing) becomes a stochastic process in its own right, and we can study its volatility properties, it turns out to have a beneficial effect. In terms of the component spin phasors, differentiating (A 8) we find

(D 1) $$P_{t}=\frac{1}{N^{1/2}}\sum_{\left(j\right)}is_{t}^{\left(j\right)}h_{t}^{\left(j\right)},$$

where we have used the relation $\text{d}s_{t}^{\left(j\right)}=is_{t}^{\left(j\right)}h_{t}^{\left(j\right)}\text{d}t$ which follows from equation (A 5). Now, computing the stochastic differential of equation (D 1), and then taking the modulus squared differential product, we find

(D 2) $$\begin{array}{rl} \text{d}P_{t}\text{d}P_{t}^{*} & =\frac{1}{N}\left\{\sum_{\left(j\right)}\left[\text{d}s_{t}^{\left(j\right)}h_{t}^{\left(j\right)}+s_{t}^{\left(j\right)}\text{d}h_{t}^{\left(j\right)}\right]\times \sum_{\left(k\right)}\left[\text{d}s_{t}^{(k)*}h_{t}^{\left(k\right)}+s_{t}^{(k)*}\text{d}h_{t}^{\left(k\right)}\right]\right\}\\ & =\frac{1}{N}\left\{\sum_{\left(j,k\right)}s_{t}^{\left(j\right)}s_{t}^{(k)*}\text{d}h_{t}^{\left(j\right)}\text{d}h_{t}^{\left(k\right)}\right\}\\ & =\kappa^{2}\beta \text{d}t, \end{array}$$

wherein (from the second to third line) we have applied the Kronecker-delta relation between the fluctuating fields $\text{d}h_{t}^{\left(j\right)}\text{d}h_{t}^{\left(k\right)}=\kappa^{2}\beta \delta_{\mathrm{Kron}}^{jk}\text{d}t$, which follows from a spin-labelled form of equation (2.4) and the fact that the Wiener processes $W_{t}^{\left(j\right)}$ driving the random fields pertaining to spins (j) are independent. The *resulting* expression is notably independent of the spin label, and this facilitates extraction of the parametric function $\kappa^{2}\beta$ from the actual observed ensemble spin process $\Sigma_{t}$ (the aforementioned MSD), and its time derivative $P_{t}$, through the stochastic squared volatility relation

(D 3) $$\frac{\text{d}P_{t}\text{d}P_{t}^{*}}{\text{d}t}=\kappa^{2}\beta .$$

An essential property of such a quantity is that, unlike the drift of an Ito process, it is *instantaneously observable*. In other words, in the present context, given a real-time signal acquisition from an ensemble of spins, this quantity is deducible from a local time portion of the gathered data, *without* recourse to an ensemble average [17]. Finally, knowledge of $\kappa^{2}\beta$ as above, combined with that of κ from the spin spectrum, implies the values of both κ and β and therefore α also from the familiar product relation. Thereby, via the second spectral moment in combination with a novel method of stochastic volatility analysis, a complete parameter estimation scheme from observed signal data is established.

For a complementary detailed perspective on stochastic volatility models applied to spin relaxation systems, the reader is referred to [3], and in the broader context of scattering from random media for related mathematical techniques, in terms of the implementation of such schemes for discrete sampled real or simulated data, see ch. 14 in [17], and also [29].
